# Scientific sexism: the gender bias in the scientific production of the Universidade de São Paulo

**DOI:** 10.11606/s1518-8787.2021055002939

**Published:** 2021-10-18

**Authors:** Livia Oliveira-Ciabati, Luciane Loures Santos, Annie Schmaltz Hsiou, Ariane Morassi Sasso, Margaret Castro, João Paulo Souza

**Affiliations:** I Universidade de São Paulo Faculdade de Medicina de Ribeirão Preto Programa de Saúde Pública São PauloSP Brasil Universidade de São Paulo. Faculdade de Medicina de Ribeirão Preto. Programa de Saúde Pública. São Paulo, SP, Brasil; II Universidade de São Paulo Faculdade de Medicina de Ribeirão Preto Departamento de Medicina Social Ribeirão PretoSP Brasil Universidade de São Paulo. Faculdade de Medicina de Ribeirão Preto. Departamento de Medicina Social. Ribeirão Preto, SP, Brasil; III Universidade de São Paulo Faculdade de Filosofia, Ciências e Letras de Ribeirão Preto Departamento de Biologia Ribeirão PretoSP Brasil Universidade de São Paulo. Faculdade de Filosofia, Ciências e Letras de Ribeirão Preto. Departamento de Biologia. Laboratório de Paleontologia. Ribeirão Preto, SP, Brasil; IV University of Potsdam Hasso Plattner Institute Digital Health Center Potsdam Germany University of Potsdam. Hasso Plattner Institute. Digital Health Center, Potsdam, Germany; V Universidade de São Paulo Faculdade de Medicina de Ribeirão Preto Departamento de Clínica Médica Ribeirão PretoSP Brasil Universidade de São Paulo. Faculdade de Medicina de Ribeirão Preto. Departamento de Clínica Médica. Ribeirão Preto, SP, Brasil

**Keywords:** Sexism, Scientific Publication Indicators, Gender Inequality

## Abstract

**OBJECTIVE:**

To investigate gender inequity in the scientific production of the University of Sao Paulo.

**METHODS:**

Members of the University of Sao Paulo faculty are the study population. The *Web of Science* repository was the source of the publication metrics. We selected the measures: total publications and citations, average of citations per year and item, H-index, and history of citations between 1950 and 2019. We used the name of the faculty member as a proxy to the gender identity. We use descriptive statistics to characterize the metrics. We evaluated the scissors effect by selecting faculty members with a high H-index. The historical series of citations was projected until 2100. We carry out analyses for the general population and working time subgroups: less than 10 years, 10 to 20 years, and 20 years or more.

**RESULTS:**

Of the 8,325 faculty members, we included 3,067 (36.8%). Among those included, 1,893 (61.7%) were male and 1,174 (38.28%) female. The male gender presented higher values in the publication metrics (average of articles: M = 67.0 *versus* F = 49.7; average of citations/year: M = 53.9 *versus* F = 35.9), and H-index (M = 14.5 *versus* F = 12.4). Among the 100 individuals with the highest H-index (≥ 37), 83% are male. The male curve grows faster in the historical series of citations, opening a difference between the groups whose separation is confirmed by the projection.

**DISCUSSION:**

Scientific production at the Universidade de São Paulo is subject to a gender bias. Two-thirds of the faculty are male, and hiring over the past few decades perpetuates this pattern. The large majority of high impact faculty members are male.

**CONCLUSION:**

Our analysis suggests that the Universidade de São Paulo will not overcome gender inequality in scientific production without substantive affirmative action. Development does not happen by chance but through choices that are affirmative, decisive, and long-term oriented.

## INTRODUCTION

“Achieving gender equality and empowering all women and girls” is the 5^th^ Sustainable Development Goal^1^. Gender inequality is the result of centuries of female oppression and the devaluation of women and has been perpetuated to the present day. The cost of this inequality is high: the loss of human capital due to less access by girls to education is estimated to reach up to 160 trillion dollars^2^. Those who manage to reach some level of education and enter the labor market suffer from significant wage inequality. Women and men who do the same task at the same time have different salary values^2^. In addition to earning less at work, they are primarily responsible for caring for the family and household chores^3^.

Within the scientific environment, the scenario is not much better. Phenomena that perpetuate gender inequality are well known. We can cite the “Matilda effect”^4^, a systematic pattern of ignoring, not recognizing, or hiding female scientists. In the labor market, diversity, not only of gender, but ethnicity, has also proved to be a good asset for survival and innovation^5^. In science, diversity seems even more critical, especially when knowledge is fragmented and scientists are increasingly specialized.

Universities tend to be environments of innovation, constituting different axes for research and sources of new knowledge^6^. Research at universities enables the development of new technologies and products and makes it possible to develop solutions to problems of social value. The mission of developing innovative solutions to problems of social relevance, which may not be relevant to the market, is vital for universities, especially public ones^6^. Brazilian public universities are part of the network of social facilities and contribute to reducing inequalities in our society. However, like other organizations, they can be permeable to structural bias and social determinants that make the Brazilian society one of the most unequal in the world^6^.

The Universidade de São Paulo (USP) is the largest Brazilian university and the one that most contributes to the country’s scientific production, reaching the best positions in national and international classifications^7^. However, even if actions are developed to promote gender equality at USP – the university is the only Latin American representative to be part of the United Nations (UN) *HeForShe* program –, the effects of structural sexism still seem to be present. Women account for approximately half of its students and 41% of its faculty. However, just over a quarter of leadership or top academic career positions are occupied by them^8^. Considering that scientific production is one of the determinants of progression in the academic career and consequently of institutional leadership, the present study investigates gender inequalities in the USP scientific production.

## METHODS

This is a descriptive-analytical study whose objective is to evaluate metrics of scientific publication by USP professors according to gender.

### Data Sources

#### DataUSP

USP is a public institution and follows data publishing rules, such as spending on salaries, equipment, and infrastructure. In addition to these data, the university has other initiatives to provide academic and administrative indicators. *DataUSP* is the integrated repository of this data, where it is possible to view and extract decision-supportive information^9^.

#### Web of Science (WoS)

Since 2012, one of USP’s services is access to professors’ citation profiles in three repositories of publication metrics: *Web of Science, Google Scholar*, and *Scopus*. Access to the Scopus system’s application programming interfaces (API) is restricted, limiting access to information in an automated way. For this reason, they were not used in the present study. *Google Scholar* has important limitations on the quality and accuracy of the information available on the platform; thus, it was also excluded^10^. Therefore, the WoS repository was chosen as a data source for the publication metrics, collected via its Publons system.

#### Study Population

USP professors registered in DataUSP, with data available on the Publons platform and names that allow allocation in the “male” and “female” categories were included in the study population. Professors whose data were inconsistent between platforms, names that did not allow gender allocation, or had no data available on the Publons platform were excluded from the analysis.

#### Variables of Interest

The scientific production indicators used were: total publications, total citations, average of citations per year and item, H-index, and historical series of citations between 1950 and 2019.

Data on time of service at USP, salaries and other benefits received in 2019 were also collected^11^ and integrated into the database. As the time of service database does not contain USP’s unique identification number, the full name of the professors was used as a variable for integrating the databases.

The gender variable was derived from the individuals’ names^a^, which is a relevant factor for gender self-determination. Thus, a public and open database of names^12^ was used that relies on the frequency of gender in several Brazilian names, generated from the 2010 Census data.

## Data Collection and Study Procedures

**Figure 1:**
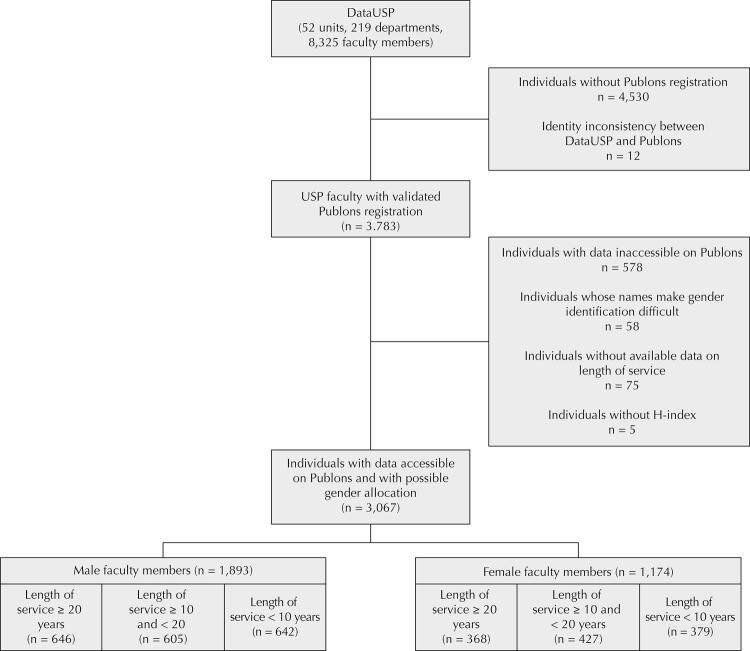
Study flowchart, 2019.

This study’s database was built from public data available on web pages, automatically extracted from API calls. All scripts used for the collection were developed in the *Python* language, and Figure 1 shows the step-by-step and the URLs of the APIs used. From the USPdigital^13^ page, professors’ names and their identifiers in the WoS were collected. A translation step was necessary from the identifiers to build the call, providing the file with interest data. In the translation stage, to ensure that the data extracted from DataUSP were consistent with the profiles found in the WoS, the profile name was also collected. The profile name and the professor’s name were compared, and those not compatible were excluded. All metrics provided by WoS were collected for each of the professors. The collection algorithm was executed on 20 Jun 2020. Although all the data used were public, once the study database was constituted, it was anonymized to protect the faculty’s privacy and make it challenging to connect the analyzed data to the individual.

## Statistical Methods

Descriptive statistics were used for each of the metrics to assess differences in the number of publications by gender. Mann-Whitney test was used, with a significance level ≤ 0.05, to verify whether the distributions have the same pattern. Individuals with a higher H-index were selected to assess whether there is a scissors effect (reduction of female presence according to career progression). According to their respective H-indexes, the individuals were ordered, and the hundredth individual index was defined as a cut-off point. All individuals with an H-index greater than or equal to the cut-off point were selected for this analysis. The hundredth highest H-index was selected in each group (male and female) to check the distance between male and female individuals. The analyses presented above were then replicated. The faculty were categorized into H-index ranges. The gender ratio in each range was calculated with 95% confidence intervals to assess each gender’s proportion according to the H-index.

USP units were re-categorized into humanities and social sciences (e.g. history and sociology), natural and applied sciences (e.g. biology and medicine), formal sciences and related applied fields (e.g. mathematics and engineering) areas to compare the gender distribution by area of knowledge. We calculated the gender distribution in the general population and the population with the highest H-indexes for each area. The historical series of citations was drawn from the average number of citations by gender. Also, the projection until the year 2100 was calculated by polynomial linear regression of 3^th^degree. All analyses were performed using the general population and three subgroups according to the time of service at USP: less than 10 years, 10 to 20 years, and 20 years or more of service.

The analyses were performed by LO-C, using the Python language and the IDE (Integrated Development Environment) Spyder for both the statistical evaluation and the creation of the graphs. AMS performed independent verification of the analysis. The code used in this analysis is published on *GitHub*^14^

As this work uses open data, with unrestricted access and made available by the institutions themselves, approval by the ethics committee was not required^15^.

## RESULTS

The data of 8,325 academics from 219 departments of 52 teaching units were included in the survey. A total of 3,783 individuals were registered on the WoS Publons platform (45.44%), of which 3,205 made their data publicly available. A total of 3,067 Individuals (36.8%) were classified as “male” or “female”, according to their names, with 1,893 male (22.74%) and 1,174 female (14.1%). Figure 1 shows the study flowchart, including the subgroups by the time of service.

Table 1 shows the scientific production indicators of USP faculty according to gender. Of the total of 3,067 records, 61.72% were classified as male and 38.28% as female. All indicators of scientific production are higher in the male population. This pattern is repeated in the subgroups, regardless of the time of service.

**Table 1:** Indicators of scientific production of the faculty at the Universidade de São Paulo by gender between 1950 and 2019 (n = 3,067).

	Male		Female		p-value (Student’s t)
	
	Mean (standard deviation)	min–max	Mean (standard deviation)	min–max	
All faculty members					
	n = 1,893 (61.72%)		n = 1,174 (38.28%)		
H-index	14.5 (11.43)	0.0–100.0	12.38 (9.16)	0.0–54.0	< 0.01
Number of publications in the *Web of Science*	67.05 (78.9)	1–694	49.67 (53.37)	1–550	< 0.01
Total citations	1,348.28 (2,656.42)	0–36.566	859.49 (1.301.2)	0–13.461	< 0.01
Average of citations per item	16.52 (38.4)	0.0–1.536.0	14.23 (14.06)	0.0–240.3	0.05
Average of citations per year	53.97 (109.53)	0.0–2,452.33	35.9 (46.59)	0.0–420.66	< 0.01

Subgroup of individuals with 20 or more years of service at USP					
	n = 646 (63.71%)		n = 368 (36.29%)		
H-index	16.89 (11.84)	0.0–74.0	15.46 (10.36)	0.0–54.0	0.05
Number of publications in the *Web of Science*	86.24 (94.91)	1–694	68.11 (63.73)	1–476	< 0.01
Total citations	1,700.99 (2,735.52)	0–36,566	1,247.33 (1,627.37)	0–13,461	< 0.01
Average of citations per item	18,8 (61,38)	0,0–1.536,0	15,14 (13,19)	0,0–146,25	0.26
Average of citations per year	58,01 (90,0)	0,0–1.462,64	43,97 (51,11)	0,0–420,66	0.01

Subgroup of individuals with 10 to 20 years of service at USP					
	n = 605 (58.62%)		n = 427 (41.38%)		
H-index	13.29 (10.74)	0.0–100.0	11.23 (8.57)	0.0–53.0	< 0.01
Number of publications in the *Web of Science*	59.6 (68.7)	1–691	43.55 (47.05)	1–550	< 0.01
Total citations	1,157.24 (2,738.76)	0–34,889	730,0 (1,206.23)	0–11,050	< 0.01
Average of citations per item	14.81 (16.01)	0.0–216.38	13.87 (16.55)	0.0–240.3	0.36
Average of citations per year	55.39 (146.12)	0.0–2,452.33	34.93 (48.33)	0.0–333.33	0.01

Subgroup of individuals with less than 10 years of service at USP					
	n = 642 (62.88%)		n = 379 (37.12%)		
H-index	13.25 (11.27)	0.0–83.0	10.69 (7.72)	0.0–36.0	< 0.01
Number of publications in the *Web of Science*	54.78 (65.52)	1–461	38.66 (43.66)	1–314	< 0.01
Total citations	1,173.4 (2,457.73)	0–26.011	628.81 (907.9)	0–6.404	< 0.01
Average of citations per item	15.83 (17.67)	0.0–224.4	13.77 (11.6)	0.0–61.65	0.04
Average of citations per year	48.57 (84.24)	0.0–736.09	29.16 (38.18)	0.0–400.25	< 0.01

Min–max: minimum–maximum; USP: Universidade de São Paulo.

Table 2 shows the scientific production indicators among USP faculty with the highest H-index. In the general population, the cut-off point for the H-index (hundredth highest H-index) was 37 and included 112 individuals. In the subgroup with 20 or more years of service at the university, the cut-off point for the H-index was 32; between 10 and 20 years, the index was 25; in the group under 10 years, the index was equal to 20. Women make up the smallest part of the stratum of individuals with the highest indices, representing only 16.96% of academics with an H-index ≥ 37, 29.25% of academics with H-index ≥ 32 and length of service of 20 years or more and with H-index ≥ 25, 28.18% between 10 and 20 years and 22.86% less than 10 years.

**Table 2:** Indicators of scientific production of academics with the highest H-index at the Universidade de São Paulo (cut-off point equivalent to the 100th individual with the highest H-index at the university in the selected population) according to gender between 1950 and 2019.

	Male		Female		p-value (Mann-Whitney)
	
	Mean (standard deviation)	min–max	Mean (standard deviation)	min–max	
All individual (H-index ≥ 37)					
	n = 93 (83.04%)		n = 19 (16.96%)		
H-index	45.88 (11.81)	37.0–100.0	42.47 (4.79)	37.0–54.0	0.22
Number of publications in the *Web of Science*	265.34 (138.43)	80–694	228.47 (122.8)	88–550	0.28
Total citations	9,552.09 (6,653.04)	3.966–36.566	7,129.21 (2,475.77)	4.501–13.461	0.12
Average of citations per item	39.79 (26.87)	10.82–216.38	35.19 (13.33)	20.09–71.35	0.47
Average of citations per year	335.17 (331.83)	67.08–2.452.33	230.42 (74.74)	128.74–420.66	0.18

Subgroup of individuals with 20 or more years of service at USP (H-index ≥ 32)					
	n = 75 (70.75%)		n = 31 (29.25%)		
H-index	39.99 (8.1)	32.0–74.0	36.77 (5.18)	32.0–54.0	0.04
Number of publications in the *Web of Science*	256.2 (140.12)	97–694	196.45 (105.7)	80–476	0.04
Total citations	6,980.81 (4,876.05)	2,588–36,566	5,000.39 (2,483.52)	2,517–13,461	0.03
Average of citations per item	29.63 (16.5)	10.82–112.51	27.94 (9.64)	10.38–50.53	0.60
Average of citations per year	213.73 (175.15)	61.98–1,462.64	154.1 (77.78)	66.24–420.66	0.07

Subgroup of individuals with 10 to 20 years of service at USP (H-index ≥ 25)					
	n = 79 (71.82%)		n = 31 (28.18%)		
H-index	32.85 (12.16)	25.0–100.0	31.74 (7.42)	25.0–53.0	0.64
Number of publications in the *Web of Science*	171.01 (107.16)	33–691	137.81 (90.09)	52–550	0.13
Total citations	5.003,52 (6.173,31)	1.703–34.889	3.693,52 (2.205,06)	1.514–11.050	0,25
Average of citations per item	30.56 (29.18)	8.38–216.38	29.13 (13.72)	13.69–71.35	0.79
Average of citations per year	232.3 (350.62)	55.49–2.452.33	143.55 (71.67)	65.83–325.0	0.17

Subgroup of individuals with less than 10 years of service at USP (H-index ≥ 25)					
	n = 81 (77.14%)		n = 24 (22.86%)		
H-index	36.23 (11.35)	25.0–83.0	29.29 (3.87)	25.0–36.0	< 0.01
Number of publications in the *Web of Science*	178.28 (91.18)	51–461	138.25 (78.13)	41–314	0.05
Total citations	5.704,74 (4.635,64)	1.771–26.011	3.085,96 (1.269,24)	1.382–6.404	0.01
Average of citations per item	33.43 (18.75)	8.92–90.48	26.44 (12.31)	12.57–61.65	0.09
Average of citations per year	199.45 (150.86)	29.79–736.09	107.42 (75.58)	39.16–400.25	< 0.01

Min–max: minimum–maximum; USP: Universidade de São Paulo.

In the group with less than 20 years of work at USP, the H-index presents a statistical difference, reaching 20 points of difference between the highest values of each gender. Other metrics with a statistical difference in this group are the number of publications, total citations, and average of citations per year. In the group between 10 and 20 years, the number of citations also shows a statistical difference. The group with less than 10 years of work, on the other hand, has a statistical difference in all metrics, except the average number of citations per item.

Table 3 shows the ratio between genders. Regardless of the assessed H-index range or time of service at USP, there is a male predominance, with a significant confidence interval. Also, the biggest differences are in the higher index ranges, regardless of the time of service at USP. The ratio of the number of men to women tends to be higher with a higher H factor. The greater inequality among all groups is observed among individuals with the highest H factor with less than 10 years of service at the university. There are 23 male and no female individuals.

**Table 3:** Gender ratio in the Universidade de São Paulo faculty by H-index range and length of service, 2019.

H-index	All individuals	Male individuals	Female individuals	Male/female ratio (95%CI)
All individuals	3.067 (100%)	1.893 (61.72%)	1.174 (38.28%)	1.62 (1.50–1.73)
> 40.00	76 (2.48%)	62 (3.28%)	14 (1.19%)	4.43 (2.69–9.3)
35.00–39.99	65 (2.12%)	49 (2.59%)	16 (1.36%)	3.06 (1.85–6.07)
30.00–34.99	102 (3.33%)	70 (3.7%)	32 (2.73%)	2.19 (1.48–3.47)
25.00–29.99	201 (6.55%)	133 (7.03%)	68 (5.79%)	1.96 (1.48–2.66)
20.00–24.99	296 (9.65%)	196 (10.35%)	100 (8.52%)	1.96 (1.55–2.52)
15.00–19.99	436 (14.22%)	277 (14.63%)	159 (13.54%)	1.74 (1.44–2.13)
10.00–14.99	602 (19.63%)	346 (18.28%)	256 (21.81%)	1.35 (1.15–1.59)
5.00–9.99	549 (17.9%)	338 (17.86%)	211 (17.97%)	1.6 (1.35–1.91)
< 5.00	740 (24.13%)	422 (22.29%)	318 (27.09%)	1.33 (1.15–1.54)

Individuals with 20 or more years of service at USP	1.014 (100%)	646 (63.71%)	368 (36.29%)	1.76 (1.54–1.99)
> 40.00	39 (3.85%)	30 (4.64%)	9 (2.45%)	3.33 (1.75–9.15)
35.00–39.99	28 (2.76%)	21 (3.25%)	7 (1.9%)	3.0 (1.44–10.16)
30.00–34.99	63 (6.21%)	40 (6.19%)	23 (6.25%)	1.74 (1.07–3.06)
25.00–29.99	99 (9.76%)	63 (9.75%)	36 (9.78%)	1.75 (1.18–2.72)
20.00–24.99	128 (12.62%)	84 (13.0%)	44 (11.96%)	1.91 (1.35–2.82)
15.00–19.99	146 (14.4%)	92 (14.24%)	54 (14.67%)	1.7 (1.23–2.43)
10.00–14.99	170 (16.77%)	104 (16.1%)	66 (17.93%)	1.58 (1.17–2.17)
5.00–9.99	165 (16.27%)	107 (16.56%)	58 (15.76%)	1.84 (1.36–2.59)
< 5.00	176 (17.36%)	105 (16.25%)	71 (19.29%)	1.48 (1.1–2.02)

Individuals with 10 to 20 years of service at USP	1.032 (100%)	605 (58.62%)	427 (41.38%)	1.42 (1.25–1.60)
> 40.00	14 (1.36%)	9 (1.49%)	5 (1.17%)	1.8 (0.64–8.42)
35.00–39.99	22 (2.13%)	17 (2.81%)	5 (1.17%)	3.4 (1.49–18.17)
30.00–34.99	14 (1.36%)	11 (1.82%)	3 (0.7%)	3.67 (1.33–inf)
25.00–29.99	60 (5.81%)	42 (6.94%)	18 (4.22%)	2.33 (1.4–4.43)
20.00–24.99	93 (9.01%)	64 (10.58%)	29 (6.79%)	2.21 (1.46–3.59)
15.00–19.99	157 (15.21%)	95 (15.7%)	62 (14.52%)	1.53 (1.12–2.14)
10.00–14.99	197 (19.09%)	102 (16.86%)	95 (22.25%)	1.07 (0.81–1.42)
5.00–9.99	198 (19.19%)	113 (18.68%)	85 (19.91%)	1.33 (1.01–1.78)
< 5.00	277 (26.84%)	152 (25.12%)	125 (29.27%)	1.22 (0.96–1.55)

Individuals with less than 10 years of service at USP	1.021 (100%)	642 (62.88%)	379 (37.12%)	1.69 (1.49–1.92)
> 40.00	23 (2.25%)	23 (3.58%)	0 (0.0%)	10.00 (1.04–22.32)
35.00–39.99	15 (1.47%)	11 (1.71%)	4 (1.06%)	2.75 (1.04–22.32)
30.00–34.99	25 (2.45%)	19 (2.96%)	6 (1.58%)	3.17 (1.45–12.78)
25.00–29.99	42 (4.11%)	28 (4.36%)	14 (3.69%)	2.00 (1.1–4.24)
20.00–24.99	75 (7.35%)	48 (7.48%)	27 (7.12%)	1.78 (1.13–2.98)
15.00–19.99	133 (13.03%)	90 (14.02%)	43 (11.35%)	2.09 (1.48–3.1)
10.00–14.99	235 (23.02%)	140 (21.81%)	95 (25.07%)	1.47 (1.14–1.93)
5.00–9.99	186 (18.22%)	118 (18.38%)	68 (17.94%)	1.74 (1.3–2.37)
< 5.00	287 (28.11%)	165 (25.7%)	122 (32.19%)	1.35 (1.07–1.72)

95%CI: 95% confidence interval; USP: Universidade de São Paulo; inf: infinite (calculation not possible).

**Figure 2:**
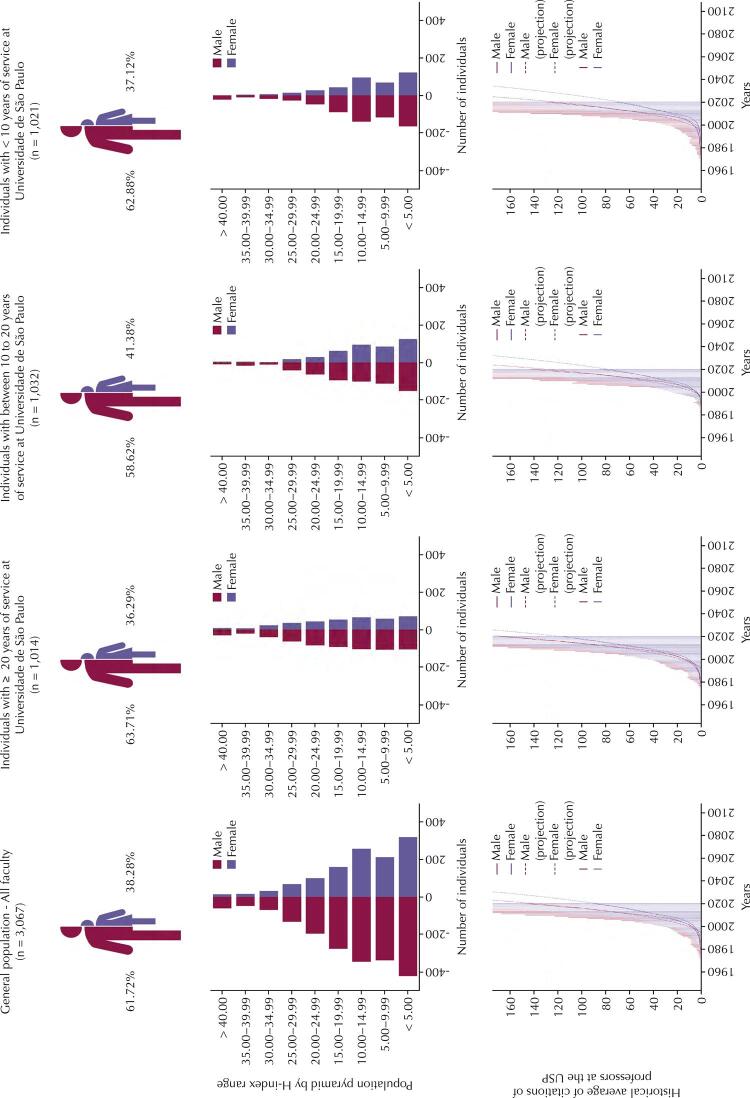
Infographic of the Universidade de São Paulo faculty, population pyramid by H-index range, and the historical average of citations with projected growth until the end of the century according to gender.

Figure 2 show the proportion of gender in the population, distribution of individuals according to the H-index range, historical average of citations, and their projection until the end of the century. The lower part of Figure 2 shows an exponential growth of the average of citations curve, with the male curve growing at a higher speed than the female curve, widening the difference between the groups over the years. The projection of these data by univariate linear regression suggests a divergent trend in the number of citations between the male and female genders. The comparative analysis of the faculty salary by gender in 2019 showed no differences (supplementary material).

## DISCUSSION

Our findings indicate that scientific production at USP is subject to gender bias. Among USP faculty who have a valid record on the most prestigious international scientific platform, the Web of Science/Publons, only a third are female. This pattern is maintained among individuals hired in the last two decades and even among those hired less than ten years ago. In general, male academics achieve more expressive scientific production metrics than female ones. The absolute majority (83.04%) of the group of individuals with high-performance scientific production, i.e., those with the highest H-indexes, is male. Our analyses also suggest that the differences in productivity between genders are not narrowing: the projection of the current trend for the coming decades indicates that the effect of gender bias on USP’s scientific production will not be overcome in the near future.

Scientific thinking has excluded and removed women since the beginning^16^. Science has historically been defined by a patriarchal, male, white, western, and financially privileged model, where men attribute reason to themselves and emotion to women. With this postulate, the ability to do science has been removed from women and attributed to men, allegedly “endowed with reason”^17^. Over time, many women who have challenged this paradigm have been ignored, minimized, and sometimes misused by their male counterparts^10^, the so-called “Matilda effect”^4^. Currently, women remain underrepresented within the scientific workforce^18^.

In Brazil, female education was neglected for 450 years, and it was only in the twentieth century that the movement to reduce the gender gap in this area started^19^. In higher education, graduates who declare themselves cisgender women are already the majority, representing 48.1% against 40.1% of graduates who declare themselves cisgender men^20^. However, even today, we observe that, as the career progresses, the proportion of women decreases and that of men increases, in a process known as the “scissor effect”^21^. Furthermore, the situation is not just numerical. The proportion of women in leadership and decision-making spaces in science is also invariably lower compared to men^22^. Our study shows that the scissors effect is perceived both in areas dominated by women^23^ and in areas with unfavorable base^18,24^. The analysis of our data confirms that the gender distribution of the 100 professors with the highest publication metrics at the university is more favorable to men, regardless of the distribution of the selected area of knowledge.

Scientific publications result from a work process beginning with the research proposal, which depends on funding to be feasible. In addition, it requires infrastructure, institutional support, and human resources. In the first stages, women are overlooked to receive the funding that makes the research feasible^25^ and work on the project^26^. Thus, since the beginning of their careers, women receive less investment and institutional support^27^, making it difficult to carry out relevant projects and, consequently, impact publications. As a parameter to progress in the academic career, the smaller number of scientific publications becomes a barrier to the progression of women in the scientific career. Our data comparing populations by the length of service at university suggests that women’s lowest number of publications begins in the first decade of their careers. There is no recovery in the following decades, contributing to enhancing the difference between genders. The *sticky floor* delays the progression of the woman’s career and can be the first step towards perpetuating gender bias when analyzing the number of publications. Corroborating this idea, a study showed that the evaluation of research projects focused only on the “quality of the proposal” presents no difference between genders, but that women lose points significantly in the evaluation of the “quality of the researcher”^28^; when removing the name of the authors, the number of articles accepted with female authors increases^29^. At the other end, women in advanced stages of their careers find it difficult to progress further, even if they have the same or even greater scientific production than their male counterparts (*glass ceiling* phenomenon)^22,30^.

Doing science is a social activity, which requires a network of contacts and collaboration between scientists. Some activities external to the institutions in which individuals are inserted can assist in forming these bonds. However, the responsibilities associated with the female gender often hinder or prevent women from participating in these networks, which are associated with better bibliometric results for both genders^31^ and allow interactions that result in greater visibility of the participants^32^, including facilitating invitations to scientific projects^33^. Within USP itself, the need to ensure women’s visibility has been discussed^34^, since the male gender was identified as a factor in selecting the press itself to choose scientific dissemination^35^. In conclusion, how scientists are treated varies according to gender and may unconsciously reaffirm the position of women as exogenous to the scientific body^36^.

Currently, there is the impression that the scenario has been changing since there is greater visibility for the problem. It has been increasingly discussed, with the proviso that the delay in the solution would be caused by problems in the structure of the university. However, two new problems must be avoided: aversion to the movement for change and the false sense of change, based on perceptions and anecdotal evidence. This perception of change can lead decision-makers to underestimate gender bias, accentuating the imbalance^37^, due to the “equality paradox” effect. Our data show that gender and publication metrics distribution has not changed in the last 20 years among professors at USP.

One of the strengths of this research was the use of an automated data collection script. This method enabled us to gather the publication data of all faculty at USP with the available identifier, collecting thousands of records for analysis. Also, when compared to the manual collection, an automatic system eliminates errors or, if it generates systematic errors, these would be present in all groups analyzed. Another strength is the data source in the university’s database, which can be considered high quality and adequate coverage of the universe analyzed. In addition to this source, bibliometrics originates from a high-quality index curated by a team of editors. The Web of Science (WoS) platform is one of the largest citation databases, with 1.7 billion citation references in more than 159 million files and 254 areas of knowledge^38^. The platform maintains strict rules to select articles for its indexes, in addition to a team of specialists who curate these items. An essential factor for the Brazilian context is the use of SciELO as a regional database integrated with WoS. As several USP research projects have a local focus and impact, ensuring that these publications are accounted for in the analysis is essential.

Among the weaknesses is the impossibility of collecting other sociodemographic information from the faculty. The analysis of gender dissociated from other social determinants is known for not explaining the whole phenomenon of inequality. It occurs especially in the ethnic-racial issue, considering that the socioeconomic conditions of black and brown women, when compared to those of white women, are worse and, consequently, the former struggle with higher levels of inequality^39^. The lack of data from individuals who did not provide their WoS identifier is another limitation. In addition, the use of the name to define gender can be criticized. However, a comprehensive database of names was used, containing the frequency of gender and consequently the Brazilian standard of denominations. While we understand that the choice of WoS as a source generates a bias in the data, it presents a higher data quality as it is a manually validated basis, despite less coverage. Finally, we recognize that scientific production is not limited to publications and citations, but these measures have a significant impact on the career development of professors.

The analysis implications of this research’s results include the need to expand studies on the expression mechanisms of sexism in the university environment and develop solutions to combat it. Our findings’ possible practical implications include discussing the implementation of systemic interventions of an affirmative and countercyclical nature, such as research grants dedicated to female researchers. The goal is to ensure minimal and adequate proportions of female representation in teaching vacancies and ensure the distribution of incentives research, particularly for women at the highest levels of their careers.

Although the Universidade de São Paulo has been developing actions to achieve gender equality, our data shows that inequalities persist and will hardly be overcome without substantive affirmative action. Adopting effective systemic actions is fundamental for the achievement of gender equality in this generation of researchers. Development does not happen by chance but through choices that are affirmative, decisive, and long-term oriented.

## Footnotes

a The authors recognize the limited way in which gender is used in this work, understanding that gender is not a binary definition and that this approach can be considered reductive. With this clarification, the terminology is used since the variable “name” is more related to self-perception and self-determination of gender than biological sex.
