# Supplementary material for: Scientific sexism: the gender bias in the scientific production of the Universidade de São Paulo
Source: Rev Saude Publica. 2021 Oct 18;55:46. doi: 10.11606/s1518-8787.2021055002939 (PMC8522718; doi:10.11606/s1518-8787.2021055002939)
Supplement: Translation

# Translation: Sexismo científico: o viés de gênero na produção científica da Universidade de São Paulo

**Authors:** Livia Oliveira-Ciabati, Luciane Loures Santos, Annie Schmaltz Hsiou, Ariane Morassi Sasso, Margaret Castro, João Paulo Souza

**Affiliations:** I Brasil Universidade de São Paulo. Faculdade de Medicina de Ribeirão Preto Programa de Saúde Pública. São Paulo, SP, Brasil; II Brasil Universidade de São Paulo. Faculdade de Medicina de Ribeirão Preto. Departamento de Medicina Social. Ribeirão Preto, SP, Brasil; III Brasil Universidade de São Paulo. Faculdade de Filosofia, Ciências e Letras de Ribeirão Preto. Departamento de Biologia. Laboratório de Paleontologia. Ribeirão Preto, SP, Brasil; IV Germany University of Potsdam. Hasso Plattner Institute. Digital Health Center, Potsdam, Germany; V Brasil Universidade de São Paulo. Faculdade de Medicina de Ribeirão Preto. Departamento de Clínica Médica. Ribeirão Preto, SP, Brasil

**Keywords:** Sexismo, Indicadores de Produção Científica, Iniquidade de Gênero

## Abstract

**OBJETIVO:**

Investigar desigualdades de gênero na produção científica de docentes da Universidade de São Paulo.

**MÉTODOS:**

A população consiste em professores(as) da Universidade de São Paulo. O repositório *Web of Science* foi a fonte das métricas de publicação. Selecionamos as medidas: total de publicações e citações, média de citações por ano e por item, índice H e histórico de citações entre 1950 e 2019. Usamos o nome do(a) docente como um proxy para a identidade de gênero. Usamos estatísticas descritivas para caracterizar as métricas. Avaliamos o efeito tesoura selecionando os(as) professores(as) com índice H alto. A série histórica de citações foi projetada até 2100. Realizamos análises para a população geral e subgrupos de tempo de trabalho: menos de 10 anos, de 10 a 20 anos e 20 anos ou mais.

**RESULTADOS:**

Dos 8.325 docentes, incluímos 3.067 (36,8%). Dentre os incluídos, 1.893 (61,7%) eram professores e 1.174 (38,28%) professoras. O gênero masculino apresentou valores mais altos nas métricas de publicação (média de artigos: M = 67,0 *versus* F = 49,7; média de citações/ano: M = 53,9 *versus* F = 35,9) e índice H (M = 14,5 *versus* F = 12,4). Entre os 100 indivíduos com maior índice H (≥ 37), 83% são do gênero masculino. Na série histórica de citações, a curva masculina cresce mais rápido, abrindo uma diferença entre os grupos com afastamento confirmado pela projeção.

**DISCUSSÃO:**

A produção científica na Universidade de São Paulo está sujeita a um viés de gênero. Dois terços do corpo docente são do gênero masculino, e as contratações das últimas décadas perpetuam esse padrão. A grande maioria dos membros com alto impacto no corpo docente é do sexo masculino.

**CONCLUSÃO:**

Nossas análises sugerem que a Universidade de São Paulo não superará a desigualdade de gênero na produção científica sem uma ação afirmativa substancial. O desenvolvimento não acontece por acaso, mas por meio de escolhas afirmativas, decisivas e de longo prazo.

## INTRODUÇÃO

“Alcançar a igualdade de gênero e empoderar todas as mulheres e meninas” é o 5º Objetivo de Desenvolvimento Sustentável^1^. A desigualdade de gênero é fruto de séculos de opressão feminina e desvalorização da mulher, sendo perpetuada até os dias atuais. O custo dessa desigualdade é alto: estima-se que a perda de capital humano pelo menor acesso de meninas à educação pode chegar a até 160 trilhões de dólares^2^. Aquelas que conseguem alcançar algum nível de escolaridade e se inserir no mercado de trabalho sofrem com uma significativa inequidade salarial. Mulheres e homens que fazem a mesma tarefa pelo mesmo tempo apresentam valores salarias diferentes^2^. Além de ganhar menos no trabalho, elas são as principais responsáveis pelo cuidado da família e tarefas domésticas^3^.

Dentro do ambiente científico, o cenário não é muito melhor. Fenômenos que perpetuam a desigualdade de gênero são bem conhecidos. Podemos citar o “efeito Matilda”^4^, um padrão sistemático de ignorar, não reconhecer ou esconder cientistas mulheres. No mercado de trabalho, a diversidade, não apenas de gênero, mas étnica, também tem se provado um bom trunfo para a sobrevivência e inovação^5^. Na ciência, a diversidade parece ainda mais importante, em especial quando o conhecimento está tão fragmentado e os cientistas cada vez mais especializados.

Universidades costumam ser ambientes de inovação, constituindo-se em eixos diferenciados para pesquisa e fontes de novos conhecimentos^6^. A pesquisa nas universidades não só possibilita o desenvolvimento de novas tecnologias e produtos, mas também torna possível o desenvolvimento de soluções para problemas de valor social. A missão de desenvolver soluções inovadoras para problemas de relevância social, que podem não ser relevantes para o mercado, é particularmente importante para as universidades, principalmente as públicas^6^. As universidades públicas brasileiras fazem parte da rede de equipamentos sociais e contribuem para a redução das desigualdades de nossa sociedade. Entretanto, assim como outras organizações, elas podem ser permeáveis aos vieses estruturais e aos determinantes sociais que fazem da sociedade brasileira uma das mais desiguais do mundo^6^.

A Universidade de São Paulo (USP) é a maior universidade brasileira e a que mais contribui para a produção científica do país, alcançando as melhores posições nas classificações nacionais e internacionais^7^. Entretanto, ainda que ações sejam desenvolvidas para promover a igualdade de gênero na USP – a universidade é a única representante latino-americana a integrar o programa *HeForShe* da Organização das Nações Unidas (ONU) –, os efeitos do sexismo estrutural parecem ainda estar presentes. Embora as mulheres correspondam a aproximadamente metade de seus estudantes e 41% de seu corpo docente, pouco mais de um quarto das posições de liderança ou de topo de carreira acadêmica são ocupadas por elas^8^. Considerando que a produção científica é um dos determinantes da progressão na carreira acadêmica e consequentemente de liderança institucional, o presente estudo investiga desigualdades de gênero na produção científica da USP.

## MÉTODOS

Trata-se de um estudo descritivo-analítico cujo objetivo é avaliar métricas de publicação científica de docentes da USP segundo o gênero.

### Fontes dos Dados

#### DataUSP

A USP é uma instituição pública e segue regras de publicização de dados, como gastos com salários, equipamentos e infraestrutura. Além desses dados, a universidade tem outras iniciativas para disponibilizar indicadores acadêmicos e administrativos. O *DataUSP* é o repositório integrado desses dados, onde é possível visualizar e extrair informações de apoio à decisão^9^.

#### Web of Science (WoS)

Disponível desde 2012, um dos serviços disponibilizados pela USP é o de acesso aos perfis de citações dos docentes em três repositórios de métricas de publicação: *Web of Science, Google Scholar* e *Scopus*. O acesso às *application programming interfaces* (API) do sistema *Scopus é* restrito, limitando o acesso à informação de forma automatizada, e por essa razão elas não foram utilizadas na presente pesquisa. O *Google Scholar* apresenta importantes limitações de qualidade e acurácia da informação disponível na plataforma; por isso, também foi excluído^10^ Sendo assim, o repositório WoS foi escolhido como fonte de dados para as métricas de publicação, coletadas via seu sistema *Publons*.

## População do Estudo

Docentes da USP cadastrados no DataUSP, com dados disponíveis na plataforma *Publons* e nomes que permitem alocação nas categorias “masculino” e “feminino” foram incluídos na população de estudo. Foram excluídos da análise docentes cujos dados apresentavam inconsistência entre as plataformas, nomes que não permitiam alocação de gênero, ou que não possuíam dados disponíveis na plataforma *Publons*.

## Variáveis de Interesse

Os indicadores de produção científica utilizados foram: total de publicações, total de citações, média de citações por ano e por item, índice H e série histórica de citações entre os anos 1950 e 2019.

Dados de tempo de serviço na USP, salários e outros benefícios recebidos no ano de 2019 também foram coletados^11^ e integrados ao banco de dados. Como o banco de tempo de serviço não contém o número de identificação único da USP, foi usado o nome completo dos professores como variável de integração dos bancos.

A variável gênero foi derivada do nome dos indivíduos^a^, que é um fator relevante para a autodeterminação do gênero. Para isso foi utilizado um banco de nomes^12^ público e aberto que conta com a frequência de gênero em diversos nomes brasileiros, gerado a partir de dados do Censo de 2010.

## Coleta de Dados e Procedimentos do Estudo

O banco de dados deste estudo foi construído a partir de dados públicos disponíveis em páginas *web*, sendo extraídos de forma automática a partir de chamadas de API. Todos os *scripts* utilizados para coleta foram desenvolvidos na linguagem *Python*, sendo que a Figura 1 apresenta o passo a passo e as URL das API utilizadas. Da página da USPdigital^13^ foram coletados os nomes dos docentes e seus identificadores no WoS. A partir dos identificadores, foi necessário um passo de tradução para construir a chamada, retornando o arquivo com os dados de interesse. Na etapa de tradução, para garantir que os dados extraídos do DataUSP fossem consistentes com os perfis encontrados no WoS, foi coletado também o nome do perfil. O nome do perfil e o nome do docente foram comparados, e aqueles que não eram compatíveis foram excluídos. Todas as métricas fornecidas pelo WoS foram coletadas para cada um dos professores. O algoritmo de coleta foi executado no dia 20 de junho de 2020. Ainda que todos os dados utilizados fossem públicos, uma vez constituído o banco de dados do estudo, ele foi anonimizado, de forma a proteger a privacidade do corpo docente e dificultar a vinculação entre os dados analisados e o indivíduo.

**Figura 1:** Fluxograma do estudo, 2019.

## Métodos Estatísticos

Para avaliar se há diferença entre o número de publicações por gênero, foi utilizada estatística descritiva para cada uma das métricas. Para verificar se as distribuições apresentam o mesmo padrão, foi utilizado o teste Mann-Whitney, com nível de significância ≤ 0,05. Para avaliar se há o efeito tesoura (redução da presença feminina conforme a progressão da carreira), foram selecionados docentes com maior índice H. A lista de professores foi ordenada de acordo com os seus respectivos índices H, e o índice do centésimo docente foi definido como ponto de corte. Todos os professores com índice H maior ou igual ao ponto de corte foram selecionados para essa análise. Para verificar a distância entre professores e professoras, foi selecionado o centésimo índice H mais alto em cada um dos grupos (masculino e feminino) e foram replicadas as análises apresentadas anteriormente. Para avaliar a proporção de cada gênero de acordo com o índice H, os docentes foram categorizados em faixas do índice H, e a razão de gênero em cada faixa foi calculada, juntamente com seus intervalos de confiança de 95%.

Para comparar a distribuição de gênero por área de conhecimento, as unidades da USP foram recategorizadas em exatas, humanas e biológicas. Para cada uma dessas áreas, calculamos a distribuição de gênero na população geral e na população com os maiores índices H. A série histórica de citações foi desenhada a partir da média de citações por gênero, e a projeção até o ano 2100 foi calculada por regressão linear polinomial de grau 3. Todas as análises foram realizadas usando a população geral e três subgrupos de acordo com tempo de serviço na USP: menos de 10 anos, de 10 a 20 anos e 20 anos ou mais de serviço.

As análises foram realizadas por LO-C, utilizando a linguagem *Python* e a IDE (*Integrated Development Environment*) *Spyder* tanto para a avaliação estatística quanto para a criação dos gráficos. AMS realizou verificação independente da análise. O código utilizado nessa análise está publicado no *GitHub*^14^

Como este trabalho utiliza dados abertos, de acesso irrestrito e disponibilizados pelas próprias instituições, não foi necessária a aprovação pelo comitê de ética em pesquisa^15^.

## RESULTADOS

Os dados de 8.325 docentes, oriundos de 219 departamentos de 52 unidades de ensino, foram incluídos na pesquisa. Um total de 3.783 docentes possuía cadastro na plataforma *Publons* da WoS (45,44%), dos quais 3.205 disponibilizavam seus dados publicamente. Um total de 3.067 docentes (36,8%) foi classificado em “masculino” ou “feminino”, segundo nome, sendo 1.893 professores (22,74%) e 1.174 professoras (14,1%). A Figura 1 apresenta o fluxograma do estudo, incluindo os subgrupos por tempo de serviço.

A Tabela 1 apresenta os indicadores de produção científica dos docentes da USP, segundo o gênero. Do total de 3.067 registros, 61,72% foram classificados no gênero masculino e 38,28% no gênero feminino. Todos os indicadores de produção científica são maiores na população masculina de docentes. Esse padrão se repete nos subgrupos de docentes, independentemente do tempo de serviço.

**Tabela 1:** Indicadores de produção científica de docentes da Universidade de São Paulo por gênero entre 1950 e 2019 (n = 3.067).

	Masculino		Feminino		p-valor (t de Student)
	
	Média (desvio-padrão)	mín–max	Média (desvio-padrão)	mín–max	
Todos os docentes					
	n = 1.893 (61,72%)		n = 1.174 (38,28%)		
Índice H	14,5 (11,43)	0,0–100,0	12,38 (9,16)	0,0–54,0	< 0,01
Número de publicações no *Web of Science*	67,05 (78,9)	1–694	49,67 (53,37)	1–550	< 0,01
Total de citações	1.348,28 (2.656,42)	0–36.566	859,49 (1.301,2)	0–13.461	< 0,01
Média de citações por item	16,52 (38,4)	0,0–1.536,0	14,23 (14,06)	0,0–240,3	0,05
Média de citações por ano	53,97 (109,53)	0,0–2.452,33	35,9 (46,59)	0,0–420,66	< 0,01

Subgrupo de professores com 20 anos ou mais de tempo de serviço na USP					
	n = 646 (63,71%)		n = 368 (36,29%)		
Índice H	16,89 (11,84)	0,0–74,0	15,46 (10,36)	0,0–54,0	0,05
Número de publicações no *Web of Science*	86,24 (94,91)	1–694	68,11 (63,73)	1–476	< 0,01
Total de citações	1.700,99 (2.735,52)	0–36.566	1.247,33 (1.627,37)	0–13.461	< 0,01
Média de citações por item	18,8 (61,38)	0,0–1.536,0	15,14 (13,19)	0,0–146,25	0,26
Média de citações por ano	58,01 (90,0)	0,0–1.462,64	43,97 (51,11)	0,0–420,66	0,01

Subgrupo de professores com 10 a 20 anos de tempo de serviço na USP					
	n = 605 (58,62%)		n = 427 (41,38%)		
Índice H	13,29 (10,74)	0,0–100,0	11,23 (8,57)	0,0–53,0	< 0,01
Número de publicações no *Web of Science*	59,6 (68,7)	1–691	43,55 (47,05)	1–550	< 0,01
Total de citações	1157,24 (2.738,76)	0–34.889	730,0 (1.206,23)	0–11.050	< 0,01
Média de citações por item	14,81 (16,01)	0,0–216,38	13,87 (16,55)	0,0–240,3	0,36
Média de citações por ano	55,39 (146,12)	0,0–2.452,33	34,93 (48,33)	0,0–333,33	0,01

Subgrupo de professores com menos de 10 anos de tempo de serviço na USP					
	n = 642 (62,88%)		n = 379 (37,12%)		
Índice H	13,25 (11,27)	0,0–83,0	10,69 (7,72)	0,0–36,0	< 0,01
Número de publicações no *Web of Science*	54,78 (65,52)	1–461	38,66 (43,66)	1–314	< 0,01
Total de citações	1.173,4 (2.457,73)	0–26.011	628,81 (907,9)	0–6.404	< 0,01
Média de citações por item	15,83 (17,67)	0,0–224,4	13,77 (11,6)	0,0–61,65	0,04
Média de citações por ano	48,57 (84,24)	0,0–736,09	29,16 (38,18)	0,0–400,25	< 0,01

Mín–max: mínimo–máximo; USP: Universidade de São Paulo.

A Tabela 2 apresenta os indicadores de produção científica entre os docentes da USP com maior índice H. Na população geral, o ponto de corte do índice H (centésimo maior índice H) foi 37 e incluiu 112 docentes. No subgrupo com 20 anos ou mais de tempo de serviço na universidade, o ponto de corte do índice H foi de 32; entre 10 e 20 anos, o índice foi 25; no grupo com menos de 10 anos, o índice foi igual a 20. As mulheres compõem a menor parte do estrato de docentes com maiores índices, representando apenas 16,96% dos docentes com índice H ≥ 37, 29,25% dos docentes com índice H ≥ 32 e tempo de serviço de 20 anos ou mais e com índice H ≥ 25, 28,18% entre 10 e 20 anos e 22,86% menor que 10 anos.

**Tabela 2:** Indicadores de produção científica de docentes com maior índice H na Universidade de São Paulo (ponto de corte equivalente ao centésimo docente com índice H mais alto da universidade na população selecionada) segundo gênero entre 1950 e 2019.

	Masculino		Feminino		p-valor (Mann-Whitney)
	
	Média (desvio-padrão)	mín–max	Média (desvio-padrão)	mín–max	
Todos os docentes (índice H ≥ 37)					
	n = 93 (83,04%)		n = 19 (16,96%)		
Índice H	45,88 (11,81)	37,0–100,0	42,47 (4,79)	37,0–54,0	0,22
Número de publicações no *Web of Science*	265,34 (138,43)	80–694	228,47 (122,8)	88–550	0,28
Total de citações	9552,09 (6.653,04)	3.966–36.566	7129,21 (2.475,77)	4.501–13.461	0,12
Média de citações por item	39,79 (26,87)	10,82–216,38	35,19 (13,33)	20,09–71,35	0,47
Média de citações por ano	335,17 (331,83)	67,08–2.452,33	230,42 (74,74)	128,74–420,66	0,18

Subgrupo de professores 20 anos ou mais de tempo de serviço na USP (índice H ≥ 32)					
	n = 75 (70,75%)		n = 31 (29,25%)		
Índice H	39,99 (8,1)	32,0–74,0	36,77 (5,18)	32,0–54,0	0,04
Número de publicações no *Web of Science*	256,2 (140,12)	97–694	196,45 (105,7)	80–476	0,04
Total de citações	6.980,81 (4.876,05)	2.588–36.566	5.000,39 (2.483,52)	2.517–13.461	0,03
Média de citações por item	29,63 (16,5)	10,82–112,51	27,94 (9,64)	10,38–50,53	0,60
Média de citações por ano	213,73 (175,15)	61,98–1.462,64	154,1 (77,78)	66,24–420,66	0,07

Subgrupo de professores com 10 a 20 anos de tempo de serviço na USP (índice H ≥ 25)					
	n = 79 (71,82%)		n = 31 (28,18%)		
Índice H	32,85 (12,16)	25,0–100,0	31,74 (7,42)	25,0–53,0	0,64
Número de publicações no *Web of Science*	171,01 (107,16)	33–691	137,81 (90,09)	52–550	0,13
Total de citações	5.003,52 (6.173,31)	1.703–34.889	3.693,52 (2.205,06)	1.514–11.050	0,25
Média de citações por item	30,56 (29,18)	8,38–216,38	29,13 (13,72)	13,69–71,35	0,79
Média de citações por ano	232,3 (350,62)	55,49–2.452,33	143,55 (71,67)	65,83–325,0	0,17

Subgrupo de professores com menos de 10 anos de tempo de serviço na USP (índice H ≥ 25)					
	n = 81 (77,14%)		n = 24 (22,86%)		
Índice H	36,23 (11,35)	25,0–83,0	29,29 (3,87)	25,0–36,0	< 0,01
Número de publicações no *Web of Science*	178,28 (91,18)	51–461	138,25 (78,13)	41–314	0,05
Total de citações	5.704,74 (4.635,64)	1.771–26.011	3.085,96 (1.269,24)	1.382–6.404	0,01
Média de citações por item	33,43 (18,75)	8,92–90,48	26,44 (12,31)	12,57–61,65	0,09
Média de citações por ano	199,45 (150,86)	29,79–736,09	107,42 (75,58)	39,16–400,25	< 0,01

Mín–max: mínimo–máximo; USP: Universidade de São Paulo.

No grupo com menos de 20 anos de trabalho na USP, o índice H apresenta diferença estatística, chegando a 20 pontos de diferença entre os maiores valores de cada gênero. Outras métricas com diferença estatística nesse grupo são o número de publicações, total de citações e média de citações por ano. No grupo entre 10 e 20 anos, o número de citações também apresenta diferença estatística. Já o grupo com menos de 10 anos de trabalho apresenta diferença estatística em todas as métricas, exceto média de citações por item.

Na Tabela 3 apresentamos a razão entre os gêneros. Independente da faixa de índice H avaliada ou tempo de serviço à USP, existe uma predominância masculina, com intervalo de confiança significativo. Além disso, as maiores diferenças são nas faixas mais altas do índice, independentemente do tempo de serviço à USP. A razão de número de homens para mulheres tende a ser maior quanto maior o fator H. A maior desigualdade entre todos os grupos é observada entre os docentes de maior fator H com menos de 10 anos de tempo de serviço na universidade, categoria em que há 23 professores e nenhuma professora.

**Tabela 3:** Proporção de gênero por faixa de índice H e tempo de serviço à Universidade de São Paulo, 2019.

Índice H	Todos os docentes	Professores	Professoras	Razão masculino/feminino (IC95%)
Todos os docentes	3.067 (100%)	1.893 (61,72%)	1.174 (38,28%)	1,62 (1,50–1,73)
> 40,00	76 (2,48%)	62 (3,28%)	14 (1,19%)	4,43 (2,69–9,3)
35,00–39,99	65 (2,12%)	49 (2,59%)	16 (1,36%)	3,06 (1,85–6,07)
30,00–34,99	102 (3,33%)	70 (3,7%)	32 (2,73%)	2,19 (1,48–3,47)
25,00–29,99	201 (6,55%)	133 (7,03%)	68 (5,79%)	1,96 (1,48–2,66)
20,00–24,99	296 (9,65%)	196 (10,35%)	100 (8,52%)	1,96 (1,55–2,52)
15,00–19,99	436 (14,22%)	277 (14,63%)	159 (13,54%)	1,74 (1,44–2,13)
10,00–14,99	602 (19,63%)	346 (18,28%)	256 (21,81%)	1,35 (1,15–1,59)
5,00–9,99	549 (17,9%)	338 (17,86%)	211 (17,97%)	1,6 (1,35–1,91)
< 5,00	740 (24,13%)	422 (22,29%)	318 (27,09%)	1,33 (1,15–1,54)

Docentes com 20 anos ou mais de tempo de serviço na USP	1.014 (100%)	646 (63,71%)	368 (36,29%)	1,76 (1,54–1,99)
> 40,00	39 (3,85%)	30 (4,64%)	9 (2,45%)	3,33 (1,75–9,15)
35,00–39,99	28 (2,76%)	21 (3,25%)	7 (1,9%)	3,0 (1,44–10,16)
30,00–34,99	63 (6,21%)	40 (6,19%)	23 (6,25%)	1,74 (1,07–3,06)
25,00–29,99	99 (9,76%)	63 (9,75%)	36 (9,78%)	1,75 (1,18–2,72)
20,00–24,99	128 (12,62%)	84 (13,0%)	44 (11,96%)	1,91 (1,35–2,82)
15,00–19,99	146 (14,4%)	92 (14,24%)	54 (14,67%)	1,7 (1,23–2,43)
10,00–14,99	170 (16,77%)	104 (16,1%)	66 (17,93%)	1,58 (1,17–2,17)
5,00–9,99	165 (16,27%)	107 (16,56%)	58 (15,76%)	1,84 (1,36–2,59)
< 5,00	176 (17,36%)	105 (16,25%)	71 (19,29%)	1,48 (1,1–2,02)

Docentes com 10 a 20 anos de tempo de serviço na USP	1.032 (100%)	605 (58,62%)	427 (41,38%)	1,42 (1,25–1,60)
> 40,00	14 (1,36%)	9 (1,49%)	5 (1,17%)	1,8 (0,64–8,42)
35,00–39,99	22 (2,13%)	17 (2,81%)	5 (1,17%)	3,4 (1,49–18,17)
30,00–34,99	14 (1,36%)	11 (1,82%)	3 (0,7%)	3,67 (1,33–inf)
25,00–29,99	60 (5,81%)	42 (6,94%)	18 (4,22%)	2,33 (1,4–4,43)
20,00–24,99	93 (9,01%)	64 (10,58%)	29 (6,79%)	2,21 (1,46–3,59)
15,00–19,99	157 (15,21%)	95 (15,7%)	62 (14,52%)	1,53 (1,12–2,14)
10,00–14,99	197 (19,09%)	102 (16,86%)	95 (22,25%)	1,07 (0,81–1,42)
5,00–9,99	198 (19,19%)	113 (18,68%)	85 (19,91%)	1,33 (1,01–1,78)
< 5,00	277 (26,84%)	152 (25,12%)	125 (29,27%)	1,22 (0,96–1,55)

Docentes com menos de 10 anos de tempo de serviço na USP	1.021 (100%)	642 (62,88%)	379 (37,12%)	1,69 (1,49–1,92)
> 40,00	23 (2,25%)	23 (3,58%)	0 (0,0%)	10,00 (1,04–22,32)
35,00–39,99	15 (1,47%)	11 (1,71%)	4 (1,06%)	2,75 (1,04–22,32)
30,00–34,99	25 (2,45%)	19 (2,96%)	6 (1,58%)	3,17 (1,45–12,78)
25,00–29,99	42 (4,11%)	28 (4,36%)	14 (3,69%)	2,00 (1,1–4,24)
20,00–24,99	75 (7,35%)	48 (7,48%)	27 (7,12%)	1,78 (1,13–2,98)
15,00–19,99	133 (13,03%)	90 (14,02%)	43 (11,35%)	2,09 (1,48–3,1)
10,00–14,99	235 (23,02%)	140 (21,81%)	95 (25,07%)	1,47 (1,14–1,93)
5,00–9,99	186 (18,22%)	118 (18,38%)	68 (17,94%)	1,74 (1,3–2,37)
< 5,00	287 (28,11%)	165 (25,7%)	122 (32,19%)	1,35 (1,07–1,72)

IC95%: intervalo de confiança de 95%; USP: Universidade de São Paulo; inf: infinito (não foi possível calcular).

A Figura 2 e as figuras suplementares 2 a 13 apresentam a proporção de gênero na população, a distribuição dos docentes segundo a faixa de índice H, a média histórica de citações e sua projeção até o fim do século. Na parte inferior da Figura 2, observa-se que a curva da média de citações apresenta crescimento exponencial, com a curva masculina crescendo em velocidade maior que a feminina, alargando a diferença entre os grupos com o passar dos anos. A projeção desses dados por regressão linear univariada sugere tendência divergente do número de citações entre os gêneros masculino e feminino. Já a análise comparativa dos salários dos professores no ano de 2019 não apresentou diferenças (material suplementar).

**Figura 2:** Infográfico da proporção de docentes na Universidade de São Paulo, pirâmide populacional por faixa do índice H e média histórica de citações com projeção de crescimento até o fim do século conforme gênero.

## DISCUSSÃO

Nossos achados indicam que a produção científica na USP está sujeita ao viés de gênero. Entre os docentes da USP que possuem registro válido na plataforma de maior prestígio científico internacional, a plataforma *Publons* da *Web of Science*, apenas um terço é do sexo feminino. Esse padrão é mantido entre os docentes contratados nas últimas duas décadas e mesmo entre aqueles contratados há menos de dez anos. De maneira geral, os professores atingem métricas de produção científica mais expressivas que as professoras. A absoluta maioria (86,5%) do grupo de docentes com produção científica de alto desempenho, isto é, aqueles com maiores índices H, é masculina. Nossas análises sugerem, ainda, que as diferenças de produtividade entre os gêneros não estão diminuindo: a projeção da atual tendência para as próximas décadas indica que o efeito do viés de gênero na produção científica da USP não será superado em um futuro próximo.

O pensamento científico tem excluído e removido mulheres desde o início^16^. A ciência foi historicamente definida por um modelo patriarcal, masculino, branco, ocidental e financeiramente privilegiado, onde homens atribuem a si a razão e às mulheres, a emoção. Com esse postulado, a capacidade de fazer ciência tem sido removida da mulher e atribuída aos homens, que seriam “dotados de razão”^17^. Ao longo do tempo, muitas mulheres que desafiaram tal paradigma foram ignoradas, minimizadas e, por vezes, usurpadas por suas contrapartes masculinas^10^, o conhecido “efeito Matilda”^4^. Atualmente, as mulheres continuam sub-representadas dentro da força de trabalho científico^18^.

No Brasil, a educação feminina foi negligenciada por 450 anos, sendo que apenas no século XX se inicia o movimento de redução do hiato de gênero nessa área^19^. No ensino superior, graduadas que se declaram mulheres cisgêneras já são maioria, representando 48,1% contra 40,1% de graduados que se declaram homens cisgêneros^20^. Entretanto, ainda hoje observamos que, conforme a carreira avança, a proporção de mulheres diminui e a dos homens aumenta, num processo conhecido como “efeito tesoura”^21^. Além disso, a situação não é apenas numérica. A proporção de mulheres em espaços de liderança e decisão na ciência também é invariavelmente menor do que a de homens^22^. Nosso estudo mostra que o efeito tesoura é percebido tanto em áreas dominadas por mulheres^23^ quanto em áreas nas quais a base é desfavorável^18,24^. A análise dos nossos dados confirma que a distribuição de gênero dos 100 docentes com as maiores métricas de publicação na universidade é mais favorável aos homens, independentemente da distribuição da área do conhecimento selecionada.

Publicações científicas são frutos de um processo de trabalho que começa com a proposta de pesquisa, a qual depende de financiamento para ser exequível. Além disso, requer infraestrutura, apoio institucional e recursos humanos. Nas primeiras etapas, as mulheres são preteridas, tanto para receber o financiamento que viabiliza a pesquisa^25^ quanto para trabalhar no projeto^26^. Dessa forma, desde o início da carreira, as mulheres recebem menos investimento e apoio institucional^27^, o que dificulta a execução de projetos relevantes e, consequentemente, de publicações de impacto. Sendo esse um parâmetro para progredir na carreira acadêmica, o menor número de publicações científicas se torna uma barreira à progressão das mulheres na carreira científica. Nossos dados comparando as populações por tempo de serviço na universidade sugerem que o menor número de publicações pelo gênero feminino começa na primeira década da carreira e que não existe uma recuperação nas décadas seguintes, contribuindo para aumentar a diferença entre gêneros. O “*sticky floor*”, ou “chão grudento”, atrasa a progressão da carreira da mulher e pode ser o primeiro passo para a perpetuação do viés de gênero quando se analisa o número de publicações. Corroborando essa ideia, um estudo mostrou que a avaliação de projetos de pesquisa focados apenas na “qualidade da proposta” não apresenta diferença entre os gêneros, mas que as mulheres perdem pontos de forma significativa na avaliação da “qualidade do pesquisador”^28^; ao retirar o nome dos autores, o número de artigos aceitos com autoras mulheres aumenta^29^. Na outra ponta, mulheres que se encontram em estágios avançados da carreira encontram dificuldades para progredir mais, ainda que tenham a mesma ou até maior produção científica que suas contrapartes masculinas (fenômeno do “*glass ceiling*” ou “teto de vidro”)^22,30^.

Fazer ciência é uma atividade social, requer uma rede de contatos e de colaboração entre cientistas. Algumas atividades externas às instituições em que os indivíduos estão inseridos podem auxiliar na formação desses vínculos. Entretanto, as responsabilidades associadas ao gênero feminino muitas vezes dificultam ou impedem as mulheres de participar dessas redes, as quais estão associadas a melhores resultados bibliométricos para ambos os gêneros^31^ e permitem interações que resultam em maior visibilidade dos participantes^32^, inclusive facilitando convites para trabalhos científicos^33^. Dentro da própria USP, a necessidade de garantir visibilidade das mulheres tem sido discutida^34^, visto que o gênero masculino foi identificado como fator de seleção da própria imprensa para escolha da divulgação científica^35^. Em conclusão, a forma de tratar os cientistas varia de acordo com o gênero, podendo, inconscientemente, reafirmar a posição da mulher como exógena ao corpo científico^36^.

Há, atualmente, a impressão de que o cenário vem mudando, uma vez que há maior visibilidade para o problema, que tem sido cada vez mais discutido, com a ressalva de que a demora na solução seria causada por problemas na estrutura da universidade. Entretanto, dois novos problemas devem ser evitados: a aversão ao movimento de mudança e a falsa sensação de mudança, com base em percepções e evidências anedóticas. Essa percepção de mudança pode levar os responsáveis por tomadas de decisão a subestimar o problema do viés de gênero, acentuando o desbalanço^37^, pelo efeito “paradoxo da igualdade”. Os nossos dados mostram que a distribuição de gênero assim como a as métricas de publicação, não mudou nos últimos 20 anos entre os professores da USP.

Um dos pontos fortes desta pesquisa foi o uso de um *script* automatizado de coleta de dados. Esse método permitiu reunir os dados de publicação de todos os professores da USP com o identificador disponível, coletando milhares de registros para a análise. Além disso, quando comparada à coleta manual, um sistema automático elimina erros ou, se gerar erros sistemáticos, esses estariam presentes em todos os grupos analisados. Outro ponto forte está na fonte de dados dos docentes, em banco de dados da própria universidade, que pode ser considerado de alta qualidade e com cobertura adequada do universo analisado. Além dessa fonte, a bibliometria tem por origem um indexador de alta qualidade, curado por uma equipe de editores. A plataforma WoS é um dos maiores bancos de dados de citação, com 1,7 bilhão de referências de citações em mais de 159 milhões de arquivos e 254 áreas do conhecimento^38^. A plataforma mantém regras restritas de seleção de artigos para seus índices, além de um time de especialistas que realiza a curadoria desses itens. Um fator importante para o contexto brasileiro é a utilização do SciELO como um banco regional integrado ao WoS. Como diversas pesquisas da USP têm foco e impacto local, garantir que essas publicações estejam contabilizadas na análise é fundamental.

Entre os pontos fracos está a impossibilidade de coletar outras informações sociodemográficas dos docentes. É sabido que a análise de gênero dissociada de outros determinantes sociais não explica todo o fenômeno da desigualdade – em especial a questão étnico-racial, considerando que as condições socioeconômicas de mulheres pretas e pardas, quando comparadas às de mulheres brancas, são piores e, consequentemente, as primeiras lutam com maiores níveis de desigualdade^39^. Outro ponto negativo é o fato de que a falta de dados dos docentes que não disponibilizaram o seu identificador WoS também altera a análise dos dados. Ademais, o uso do nome para definição do gênero pode sofrer críticas. Entretanto, foi utilizada uma ampla base de dados de nomes, contendo a frequência de gênero e consequentemente o padrão brasileiro de denominações. Entendemos também que a escolha do WoS como fonte gera um viés nos dados, porém, por ser uma base validada manualmente, apresenta uma qualidade de dados maior, apesar de menor cobertura. Por último, reconhecemos que a produção cientifica não se limita a publicações e citações, porém essas medidas apresentam um impacto significativo na progressão da carreira dos docentes.

As implicações da análise dos resultados desta pesquisa incluem a necessidade de ampliar os estudos sobre os mecanismos de expressão do sexismo no ambiente universitário e o desenvolvimento de soluções que permitam o seu combate. Possíveis implicações práticas de nossos achados incluem a discussão da implementação de intervenções sistêmicas, de natureza afirmativa e anticíclica, como editais de pesquisa, para assegurar tanto proporções mínimas e adequadas de representação feminina em termos de vagas docentes quanto garantir a distribuição de incentivos de pesquisa, particularmente para mulheres nos níveis mais altos da carreira.

Embora a Universidade de São Paulo venha desenvolvendo ações para o alcançar a igualdade de gênero, nossos dados mostram que as desigualdades persistem e dificilmente serão superadas sem uma ação afirmativa substancial. A adoção de ações sistêmicas eficazes é fundamental para o alcançar a igualdade de gênero nesta geração de pesquisadores. O desenvolvimento não acontece por acaso, mas por meio de escolhas afirmativas, decisivas e de longo prazo.

## Footnotes

a Os autores reconhecem a forma limitada como gênero é utilizado neste trabalho, entendendo que gênero não é uma definição binária e que essa abordagem pode ser considerada redutiva. Com esse esclarecimento, a terminologia é usada uma vez que a variável “nome” está mais relacionada à autopercepção e autodeterminação do gênero do que do sexo biológico.
